# ImmunoGlobulin galaxy (IGGalaxy) for simple determination and quantitation of immunoglobulin heavy chain rearrangements from NGS

**DOI:** 10.1186/s12865-014-0059-7

**Published:** 2014-12-13

**Authors:** Michael J Moorhouse, David van Zessen, Hanna IJspeert, Saskia Hiltemann, Sebastian Horsman, Peter J van der Spek, Mirjam van der Burg, Andrew P Stubbs

**Affiliations:** Department of Immunology, Erasmus University Medical Center, Rotterdam, The Netherlands; Ontario Institute for Cancer Research, MaRS Centre, Toronto, Ontario Canada; Department of Bioinformatics, Erasmus University Medical Center, Rotterdam, The Netherlands; Department of Experimental Urology, Erasmus MC, Erasmus University Medical Center, s-Gravendijkwal 230, 3015 CE Rotterdam, The Netherlands

**Keywords:** Next generation sequencing, Immunoglobulin heavy chain, Repertoire, IMGT/HIGHV-QUEST, igBLAST

## Abstract

**Background:**

Sequence analysis of immunoglobulin heavy chain (IGH) gene rearrangements and frequency analysis is a powerful tool for studying the immune repertoire, immune responses and immune dysregulation in health and disease. The challenge is to provide user friendly, secure and reproducible analytical services that are available for both small and large laboratories which are determining VDJ repertoire using NGS technology.

**Results:**

In this study we describe ImmunoGlobulin Galaxy (IGGalaxy)- a convenient web based application for analyzing next-generation sequencing results and reporting IGH gene rearrangements for both repertoire and clonality studies. IGGalaxy has two analysis options one using the built in igBLAST algorithm and the second using output from IMGT; in either case repertoire summaries for the B-cell populations tested are available. IGGalaxy supports multi-sample and multi-replicate input analysis for both igBLAST and IMGT/HIGHV-QUEST. We demonstrate the technical validity of this platform using a standard dataset, S22, used for benchmarking the performance of antibody alignment utilities with a 99.9 % concordance with previous results. Re-analysis of NGS data from our samples of RAG-deficient patients demonstrated the validity and user friendliness of this tool.

**Conclusions:**

IGGalaxy provides clinical researchers with detailed insight into the repertoire of the B-cell population per individual sequenced and between control and pathogenic genomes. IGGalaxy was developed for 454 NGS results but is capable of analyzing alternative NGS data (e.g. Illumina, Ion Torrent). We demonstrate the use of a Galaxy virtual machine to determine the VDJ repertoire for reference data and from B-cells taken from immune deficient patients. IGGalaxy is available as a VM for download and use on a desktop PC or on a server.

**Electronic supplementary material:**

The online version of this article (doi:10.1186/s12865-014-0059-7) contains supplementary material, which is available to authorized users.

## Background

**Figure 1:**
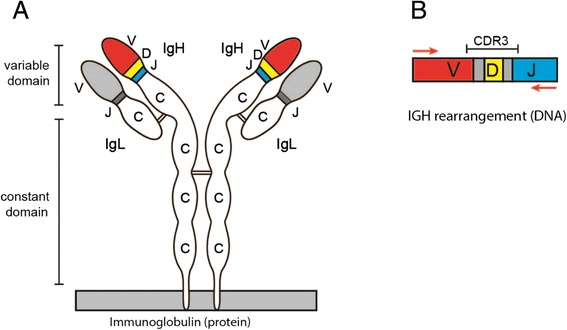
**Diagram of the Immunoglobulin Heavy chain and the regions that are sequenced. (A)** Schematic representation of an immunoglobulin protein. The V, D and J part form the variable domain and the C part forms the constant domain of the protein. **(B)** IGH rearrangements are amplified using a forward primer in the V gene and a reverse primer in the J gene. The CDR3 region consisting of part of the V gene, the D gene and part of the J gene is indicated. The red arrows represent the region that is sequenced by 454.

B lymphocytes recognize pathogens (antigens) with an antigen-specific receptor, also called antibody or immunoglobulin. This immunoglobulin is unique for every B lymphocyte, and consists of a variable and a constant domain. The locus encoding for immunoglobulin contains many different variable (V), diversity (D), and joining (J) genes. The recombination of one V and J gene, or one V, D and J gene results in the variable domain of the immunoglobulin (Figure 1A) [1] which when combined with additional processing of the gene junctions results in a potential repertoire of more than 10^12^ different immunoglobulins. However, individuals with defects in the generation of the immunoglobulins have a more restricted immunoglobulin repertoire, and are therefore vulnerable for infections.

The immunoglobulin repertoire can be studied by sequencing the rearrangements in the immunoglobulin heavy chain (IGH) locus. The use of next generation sequencing technology has increased the number of IgH rearrangements that can be assessed, but has also increased the amount of raw data requires technical knowledge to analyze. To ensure accurate and reproducible determination of immunoglobulin repertoire within and between clinical laboratories thus requires standardized and reproducible data analysis. Analysis of IGH rearrangements is complicated since potentially all sequence reads contain a unique combination of a V, D and J gene, and stretches of nucleotides that are randomly added or deleted at the V-D and D-J junctions which is spanned by the CDR3 region (Figure 1B).

Previously, bioinformatics tools have been developed that are able to align the IGH rearrangement to the reference sequences. Most frequently used tools are the ImMunoGeneTics (IMGT)/HighV-Quest [2], and IgBLAST [3]. These tools provide detailed information on the rearrangement, including the specific V, D and J genes and the composition of the junctions however information extraction and visualization of these data must be completed by the user.

## Implementation

To deliver a client and a server web-based immunoglobulin repertoire analysis and reporting tool required two implementation efforts. First the necessary analytical and reporting tools had to be implemented and the second was to implement a web-based application capable of use on and individual PC and on a server. The analytical reporting tools were integrated into the Galaxy framework (http://galaxyproject.org) [4,5] as separate tools and then as ‘end to end’ workflows to utilize Galaxy’s graphical user interface (GUI) [6]. To enable IGGalaxy to be deployed more easily on both a local PC and a server all the dependencies required to run IGGalaxy were combined into a standalone VMware (http://www.vmware.com) virtual machine (VM) - a pre-packaged environment that is used like any physical computer [7] but can be downloaded ‘ready-to-run’.

### Components for web-based analysis and visualisation

**Figure 2:**
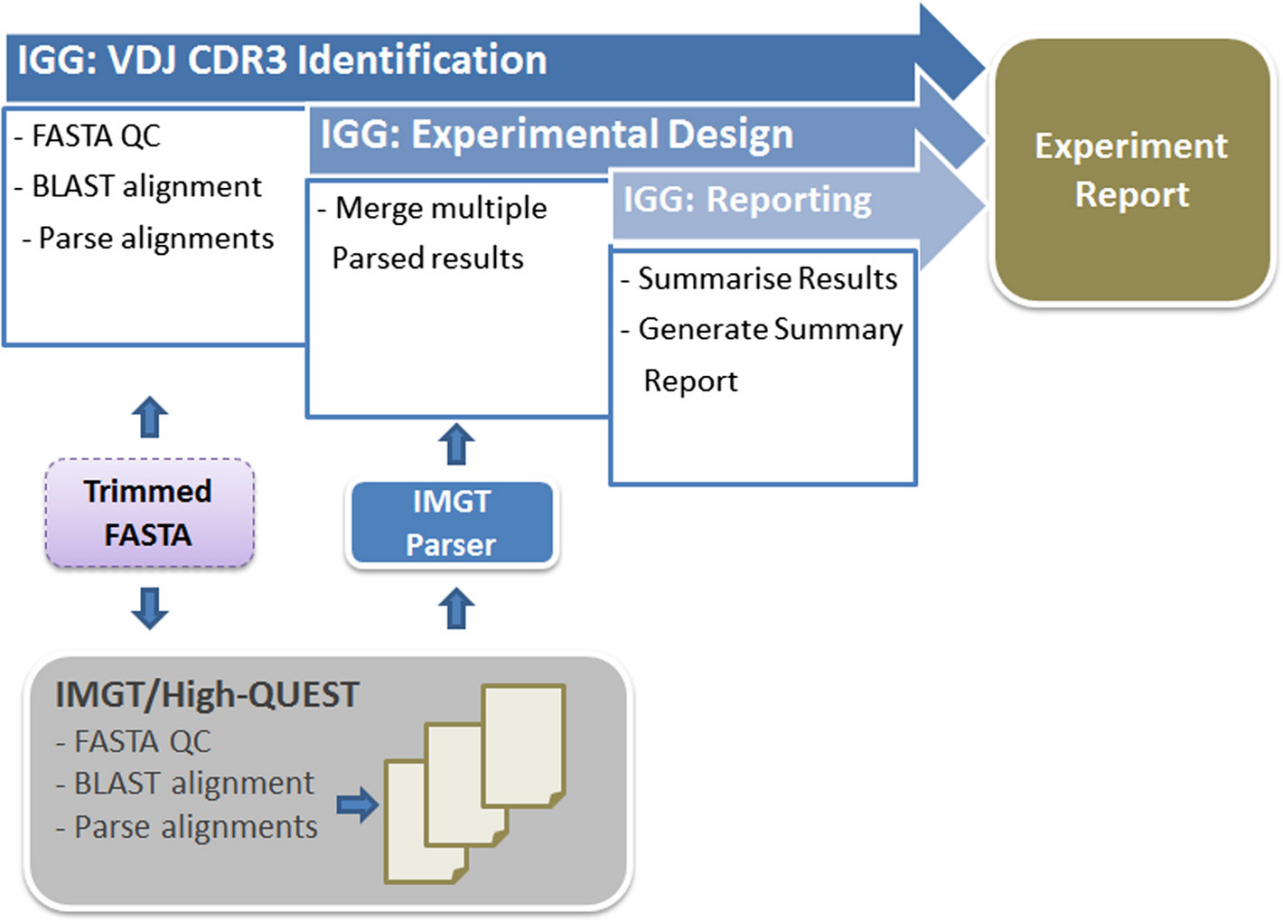
**Outline of IGGalaxy repertoire analysis and reporting.** Starting at the “Trimmed FASTA”, the user can use IMGT/HIGH-QUEST (Down) or igBLAST (Up) to perform the alignment of the sequences for further analysis in IGGalaxy. When alignment is done, the user uploads the IMGT/HIGH-QUEST result and converts it into the proprietary format or uses the igBLASTparser IGGalaxy tool to do the same for the igBLAST output. After that, the remaining steps are the same for both (or any other alignment tool that is implemented). The Experimental Design step combines several samples together for easy analysis. Finally the Reporting tool generates a report on the experiment.

A set of Perl, Python and R components have been developed to generate the framework components which include: (1) igBLAST service; (2) igBLAST parser; (3) IMGT Loader; (4) ExperimentalDesign and (5) Results summarizer and reporting (Figure 2). All tools and their respective functional description are summarized in Table 1. Instructions for installation and use of IGGalaxy are available at the project website (http://bioinformatics.erasmusmc.nl/wiki/index.php/Immunoglobulin_Galaxy).

**Table 1:** **List of tools and two workflows (igBLAST- or IMGT-ImmuneRepertoire) implemented in IGGalaxy to analyse the IgH repertoire**

**Tool name**	**Description**
igBLASTn	Galaxy wrapper for the igBLASTn executable
igBLAST Report Parser	Parsing igBLASTn report
IMGT Loader	Converting IMGT output (a zip file) into our proprietary format.
Experimental Design	Merging multiple BLAST or IMGT/H files and creating an experimental design
Report	Plotting the merged data Plotting the merged data Creating a graph of the merged reports
igBLASTn ImmuneRepertoire	Automatically runs all the tools needed for a repertoire analysis with igBLASTn
IMGT ImmuneRepertoire	Automatically runs all the tools needed for a repertoire analysis with output from IMGT (zip files).
IMGT Convert	Converting the 1_, 5_ and 6_ files

All tools with the name including BLAST or IMGT are used for analysis of igBAST and IMGT respectively. The remaining tools work for both igBLAST and IMGT.

### IGGalaxy VDJ CDR3 identification

FASTA reads must be preprocessed prior to analysis for tag removal and by trimming for low quality reads either prior to loading into IGGalaxy or by utilizing existing tools in Galaxy and IGGalaxy. FASTA sequences for all samples and replicates can be uploaded to the server quality checked, and are then ready for further analysis in IGGalaxy.

**Figure 3:**
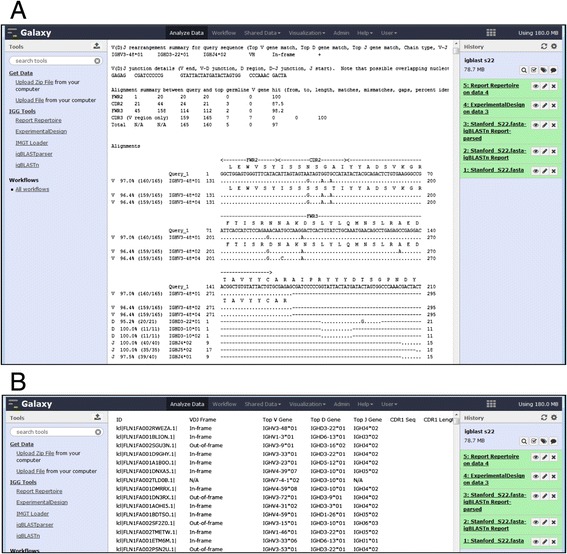
**IGGalaxy view of BLAST output in human and machine readable format. (A)** A screenshot of the report generated by igBLAST inside Galaxy, the information needed is easy to read for a human, but not for computers, **(B)** shows the same report parsed by igBLASTparser, resulting in a tabular format with all the required data for the other IGGalaxy tools stored on a separate row.

The identification of the CDR3 region and corresponding V, D, and J chains from the submitted FASTA sequences is achieved in IGGalaxy either with igBLAST or using the external IMGT/High-V-QUEST server. The igBLAST service (igBLAST) and corresponding human BLAST database (ftp://ftp.ncbi.nih.gov/blast/executables/igblast/release/) have been implemented in IGGalaxy. The standardized output using version 2.2.0.7 of igBLAST is delivered by wrapping igBLASTn with default parameters and configured to use the human reference database for germline V(D)J alignment. The output from the igBLAST service is extracted using a purpose built BLAST parser (igBLASTreport parser) designed to extract the V,D,J and CDR3 regions and convert them into the IGGalaxy feature format (Figure 3 and Additional file 1: Table S1).

In addition the user can submit their FASTA sequences to the IMGT/High-V-QUEST service (http://www.imgt.org/) [2] which has complementary features to igBLAST. The standard IMGT/High-V-QUEST analysis delivers eleven output files, a description of which is provided at http://www.imgt.org/IMGT_vquest/share/textes/imgtvquest.html#output3. To allow our users access to this content in as the alternative input to igBLAST in IGGalaxy we developed a Python loader (Upload Zip File) which transfers the compressed IMGT/High-V-QUEST output to IGGalaxy. In addition a Python parser (IMGT Loader) extracts the contents from specific files that contain the columns needed to form the proprietary format (the “1_Summary”, “5_AA-sequence” and “6_Junction” files). The parsed output from IMGT/High-V-QUEST uses common definitions with the igBLAST ReportParser (Additional file 1: Table S1) which thus normalizes the data for further analysis with IGGalaxy (Additional file 1: Table S2).

### IGGalaxy experimental design

This module allows the user to combine the parsed igBLAST results or the IMGT/High-V-QUEST with an experimental design (i.e. name samples and replicates) prior to calculation and reporting of the resultant IGH repertoires frequencies. The merger tool (ExperimentalDesign) is a python module which accepts either the parsed igBLAST file or the parsed IMGT/High-V-QUEST files and outputs a standardized file, which is required for IGGalaxy Reporting-(see Additional file 1: Tables S1 and S2). This feature of IGGalaxy allows users to utilize both formats for repertoire analysis and provides IGGalaxy extensible and backward compatibility with igBLAST and IMGT/High-V-QUEST should requirements change.

To allow the output of other annotation tools - in addition to igBLAST and IMGT - to be used with this pipeline, a clear definition of the data format has been created (Additional file 1: Table S1).

### IGGalaxy reporting

IGGalaxy provides a flexible web based analysis, viewing and downloading of results, and further analysis options using existing Galaxy genomic and statistical analysis functionality via the Report tool.

The Report tool uses a combination of several columns to determine if a sequence is functional (i.e. not out of frame or in frame with a stop codon and the CDR3 region identified) when using igBLAST and alternatively the “unproductive” annotation supplied by IMGT/HighV-QUEST.

**Figure 4:**
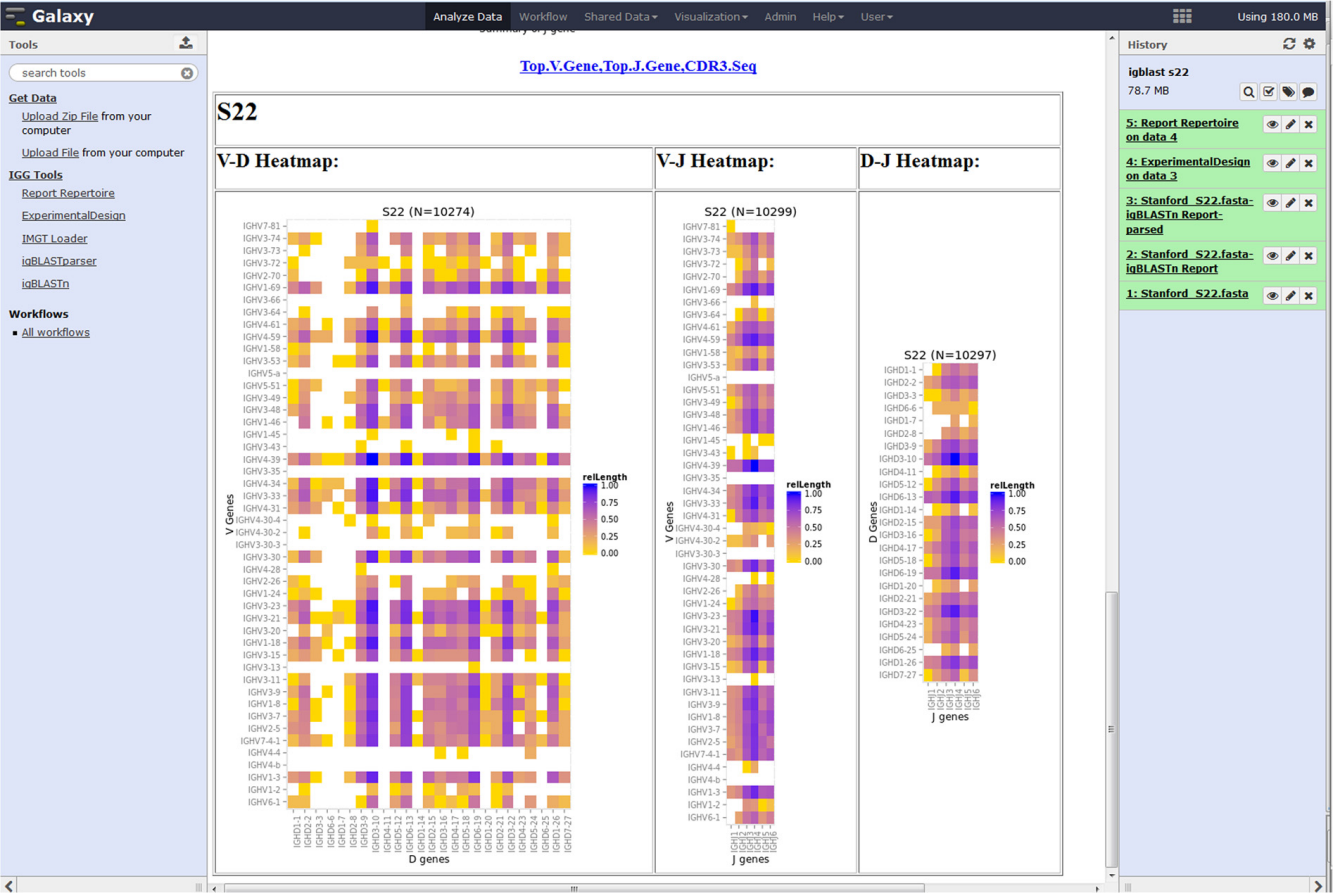
**igReport output for S22 igBLAST repertoire analysis.** The heatmaps represent the frequency of V-D, V-J and D-J combination for the individual. The “Top.V.Gene,Top.J.Gene,CDR3Seq” at the top is the clonal type definition chosen by the user when this result was generated, it also provides a downloadable link to the data used to generate the graphs in a convenient CSV format.

The output is presented in an HTML report page which summarizes the frequency of V, D, and J chains as bar charts as well as the combination V-D, V-J and D-J heatmaps based on the definition of unique sequences (e.g. V-J-CDR3 amino acid) selected in the drop down menu (Figure 4). A hyperlink embedded in the HTML report page provides the user with access to the underlying sequence results from which these plots were generated. The categories include the sample identifier as defined by the user, the unique sequence identifier relating to the original FASTA input files, the class for V-DV-J & D-J-and associated CDR (1, 2 and 3) regions and if the V-D-J, class was found in both nucleotide and protein sequences (Figure 4). The distribution of colors from yellow (low counts) to purple (high counts) normalized within a sample represent a visual cue to the variation on V, D and J repertoire for each individual sample analyzed (Figure 4).

### “End to end” analysis workflows

The implementation of the VDJ CDR3 identification, Experimental Design and Reporting in Galaxy provides the capability to chain components together in a workflow. The two Immune Repertoire analysis workflows for both igBLAST and IMGT input provide the user with an “end to end” analysis from data upload to reporting. Note that the QC component remains separate and should still be performed prior to using either of these workflows.

## Results and discussion

### Evaluation of searching method

**Figure 5:**
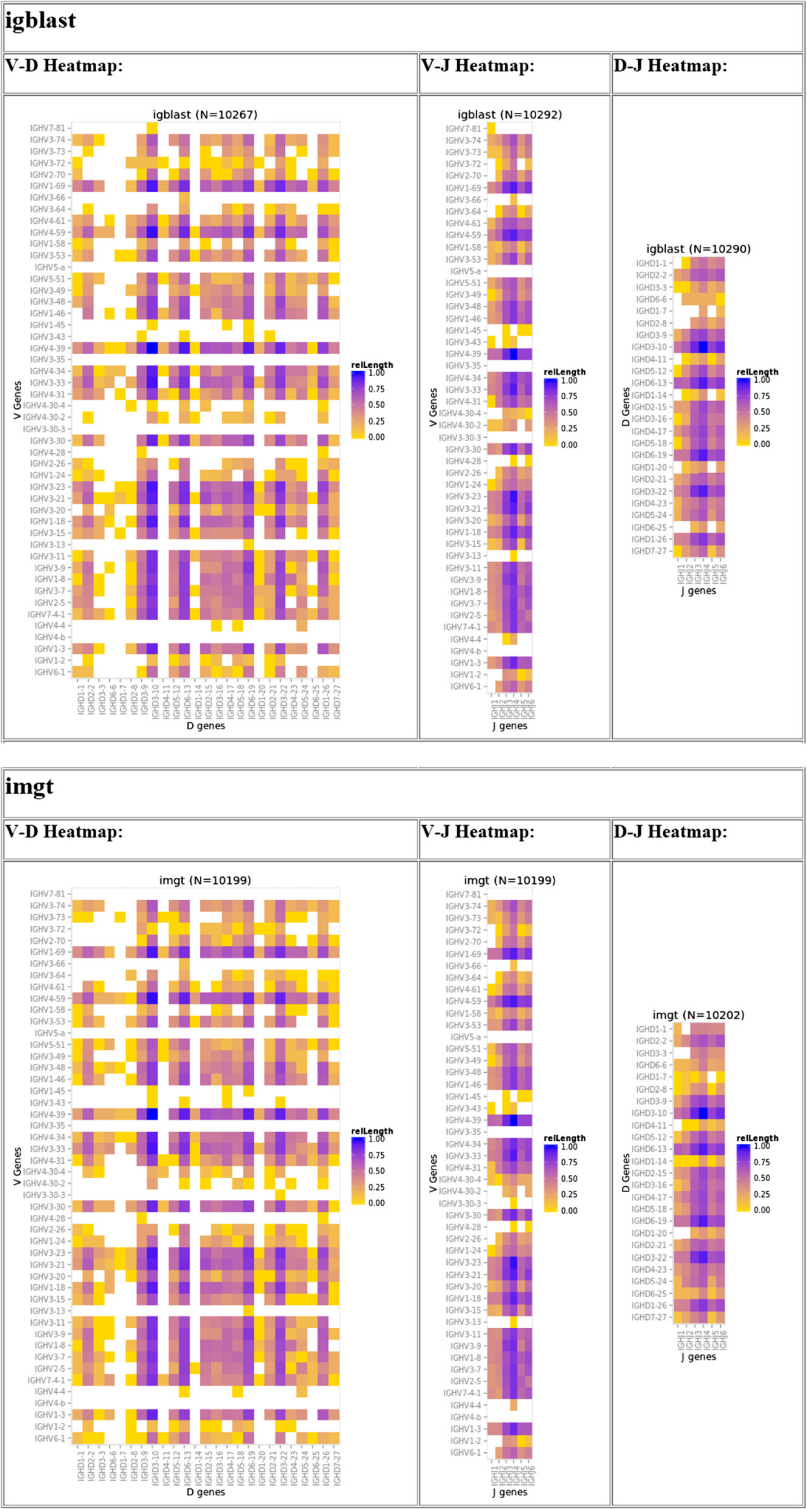
**igReport output for S22 igBLAST repertoire analysis.** The scaled frequency count (range 0 to 1) of clonal types for each V, D and J combination are represented with a heatmap which range from maximum (purple) to minimum (yellow) frequency with white representing those clonal types that are absent from IMGT or igBLAST analysis.

IMGT/High-V-QUEST and igBLAST are two pattern matching algorithms used to determine the IgH repertoire from long read NGS data (e.g. 454 reads) of S22 which has been used to demonstrate the validity of IGH VDJ assignment by several alignment utilities [8]. The S22 genotype [8] was used as the gold standard for subsequent calculation. We utilized the functionality within IGGalaxy to process both the IMGT and the parsed igBLAST results and to determine similarity of VDJ recombination detection obtained by both methods. The output of both workflows can be summarized in a single view to compare the frequency of V, D and J recombination as determined by each application using the igReport functionality (Figure 5).

The majority of VDJ recombination events determined for S22 with IGGalaxy achieved 99.9% similarity to the published genotype of S22 according to Jackson et al [8] (supplementary data) whilst the remaining 0.1% composed mainly of V4-61 or V4-59 assignment represented by 249 of the 283 sequences (Table 2). The comparison between IGGalaxy output and that of Jackson et al [8] was achieved by matching the sequences based on their VDJ identification and then determining similarity using the sequence read identifier number.

**Table 2:** **Assignment of VDJ by either the IMGT or igBLAST for the 283 sequences which are not assigned as the same VDJ compared with the S22 genotype**

**Method**	**IGHV**	**IGHD**	**IGHJ**	**Total**
IMGT	0.48	5.45	0.00	5.93
igBLAST	0.75	4.55	0.40	5.70

The values represent the % errors compared to the total number of reads assigned to the golden standard S22 genotype.

### Case study: RAGD project

We used IGGalaxy to characterize the IgH repertoire related to the immunological mechanisms causing the clinical spectrum of recombination-activating gene (RAG) deficiency (RAGD) [9]. Mutations in the recombination activating genes 1 (RAG1) and RAG2 result in loss or reduction of V(D)J recombination. In those patients that had detectable peripheral B cells we studied the IGH V(D)J recombination repertoire. IgH gene rearrangements were amplified from mononuclear cells derived from peripheral blood (PB) and/or bone marrow (BM), subsequently sequenced using next generation sequencing in healthy controls (PB and BM) and in three patients with combined immunodeficiency (CID) [9].

We previously analyzed these samples using IMGT/High-V-QUEST in combination with excel to generate the V(D)J usage frequencies [9]. The output from this reanalysis but using the IGGalaxy resulted in a similar trend to that found by Ijspeert [9], which is for control samples to have more unique sequences (Table 3).

**Table 3:** **Repertoire analysis of the RAGD dataset from IJspeert et al.** [9]

**Samples**	**All sequences**	**Unique sequences**	**Unproductive**	**Productive**
Control BM	35800	18553 (51.8)	9198 (25.7)	26602 (74.3)
P16 BM	12166	3282 (27)	2812 (23.1)	9354 (76.9)
Control PB	18901	11912 (63)	5043 (26.7)	13858 (73.3)
P15 PB	16080	7667 (47.7)	2433 (15.1)	14375 (89.4)
P16 PB	14543	3712 (25.5)	2126 (14.6)	12417 (85.4)
P18 PB	25093	3656 (14.6)	4421 (17.6)	20672 (82.4)

Unproductive = non- functional sequences and productive = functional sequences as determined with IgBLAST. PB = peripheral blood, BM = bone marrow, control = unaffected individual.

**Figure 6:**
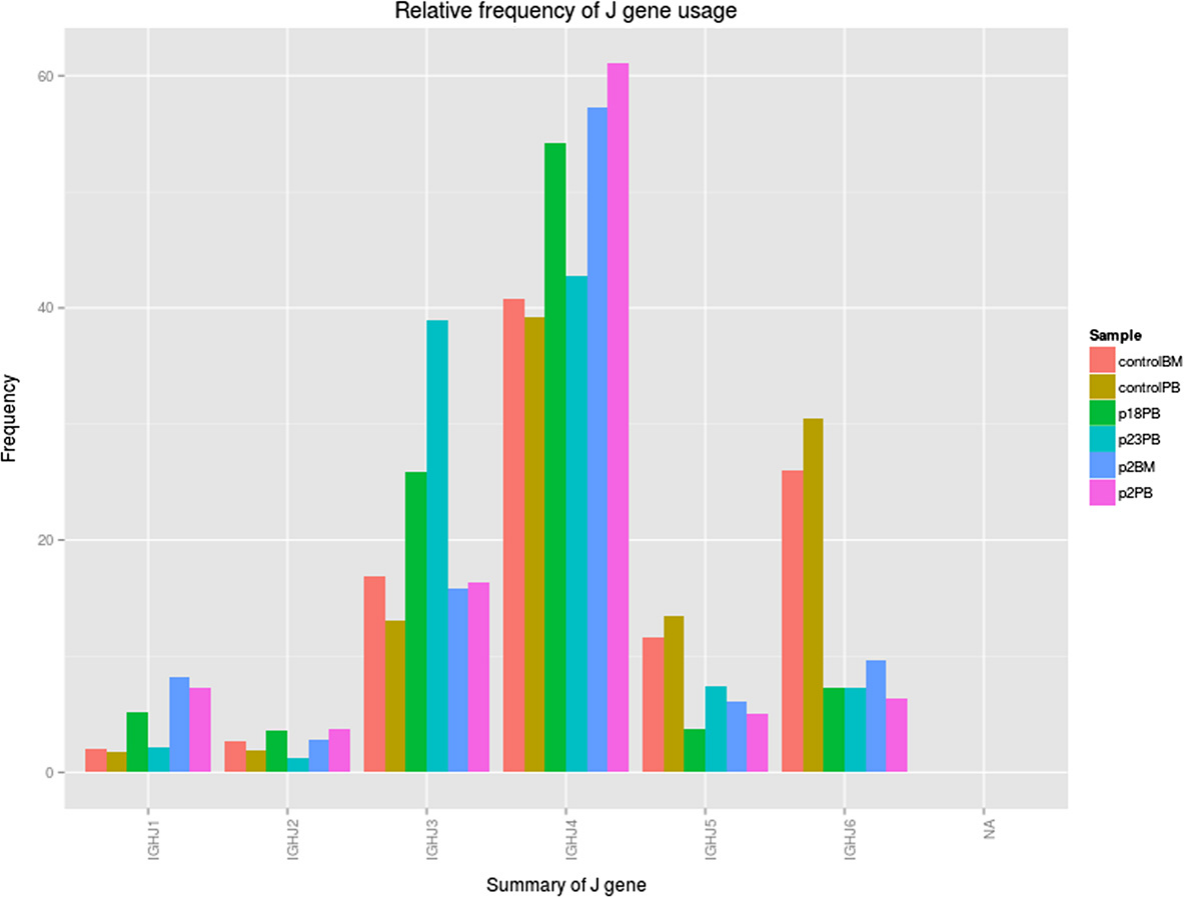
**igReport output for reanalysis of data from Ijspeert et al. [** 9 **].** A sample view two control (Control BM, Control PB), one bone marrow (P16BM) and three peripheral blood samples (P15PB, P16PB, P18PB).

The distribution of V, D and J regions as determined by the igBLAST ImmunoRepertoire function in IGGalaxy is shown as a summary between individuals in a bar chart (Figure 6) and also as a heatmap. There is clear difference between the J gene usage in the control population as compared with the RAGD patients sampled from either peripheral blood or from the bone marrow.

### Performance

We tested IGGalaxy VM using one sample (3.37 Mb, 13153 sequences) on a dual socket X5550 running at 3Ghz with 24GB of ram; to represent a more realistic environment for a standard PC the IGGalaxy VM was allocated 1 core and 1GB of memory. The igBLASTn component is the rate limiting step in the workflow (Table 4) which can be improved by assigning more cores to execute IgBLAST code with an optimal number between 4 to 8 cores (unpublished observations).

**Table 4:** **The performance of individual IGGalaxy components**

**IGGalaxy tool**	**Average time (seconds)**	**Sequences per second**
igBLASTn	604	21
igBLASTn parser	14	939
IMGT Loader	9	1461
Experimental Design	8	1644
Report Repertoire	12	1096

## Conclusions

In summary IGGalaxy provides the immunologist with a user friendly standardized client (or server) application to study repertoire analysis by two methods, igBLAST and IMGT/HighV-QUEST. IGGalaxy simplifies pre- and post-processing of FASTA sequences based on user defined clonal type selection and providing extensive visual reporting of the repertoire and associated downloadable report.

We have demonstrated accuracy of determining clonal types using IGGalaxy with the S22 data used as a golden standard and the added value of IGGalaxy for accuracy and user friendliness in re-analysis of the previously determined repertoires for RAGD patients [9]. To achieve these results we have implemented several open source services (including – of primary importance igBLAST parser, and IGGalaxy VM) which are freely available for download and for use by the research community to support immune repertoire analysis.

The common format for parsed BLAST and imported IMGT/HighV-QUEST results by IGGalaxy allows users to utilize both formats for repertoire analysis and provides IGGalaxy with future backward compatibility for both igBLAST and IMGT/High-V-QUEST and to extend the functionality of IGGalaxy beyond its current capability.

## Availability and requirements

Project name: ImmonoGlobulin galaxy

Availability: http://bioinformatics.erasmusmc.nl/wiki/index.php/Immunoglobulin_Galaxy

Operating system: Linux running in a virtual machine, VMWare, which is available for Windows, Linux and Mac OS. The virtual machine requires VMware player (https://my.vmware.com/web/vmware/free#desktop_end_user_computing/vmware_player/6_0).

Other Dependencies: The web application is implemented independently of operating system and has been successfully tested with Microsoft Internet Explorer 11.0 and Firefox 2/3 (under different versions of Linux, Microsoft Windows).

Programming Language: Python (IGGalaxy), Perl (igBLAST parser), R (IGGalaxy)

License: None required

Any restrictions to use by non-academics: No

## Additional file

Additional file 1: Table S1. Selected columns extracted from the igBLASTn output which is the base IGGalaxy Reporting Format. Column contents describe the features in every column. **Table S2.** Selected columns extracted from the files 1_Summary file, 5_AA-sequence file and 6 _Junction file from the IMGT. The Column name is the associated feature present in the IGGalaxy base Report file format generated by IGGalaxy igBLAST.

## Abbreviations

BLAST: Basic local alignment and search tool
RAG: Recombination-activating gene
RAGD: Recombination-activating gene deficiency
VM: Virtual machine
IMGT/HighV-Quest: ImMunoGeneTics (IMGT)/HighV-Quest
CID: Combined immunodeficiency
