# Supplementary material for: ImmunoGlobulin galaxy (IGGalaxy) for simple determination and quantitation of immunoglobulin heavy chain rearrangements from NGS
Source: BMC Immunol. 2014 Dec 13;15:59. doi: 10.1186/s12865-014-0059-7 (PMC4282729; doi:10.1186/s12865-014-0059-7)
Supplement: Additional file 1: Table S1. — Selected columns extracted from the igBLASTn output which is the base IGGalaxy Reporting Format. Column contents describe the features in every column. Table S2. Selected columns extracted from the files 1_Summary file, 5_AA-sequence file and 6 _Junction file from the IMGT. The Column name is the associated feature present in the IGGalaxy base Report file format generated by IGGalaxy igBLAST. [file 12865_2014_59_MOESM1_ESM.pptx]

## Slide 1
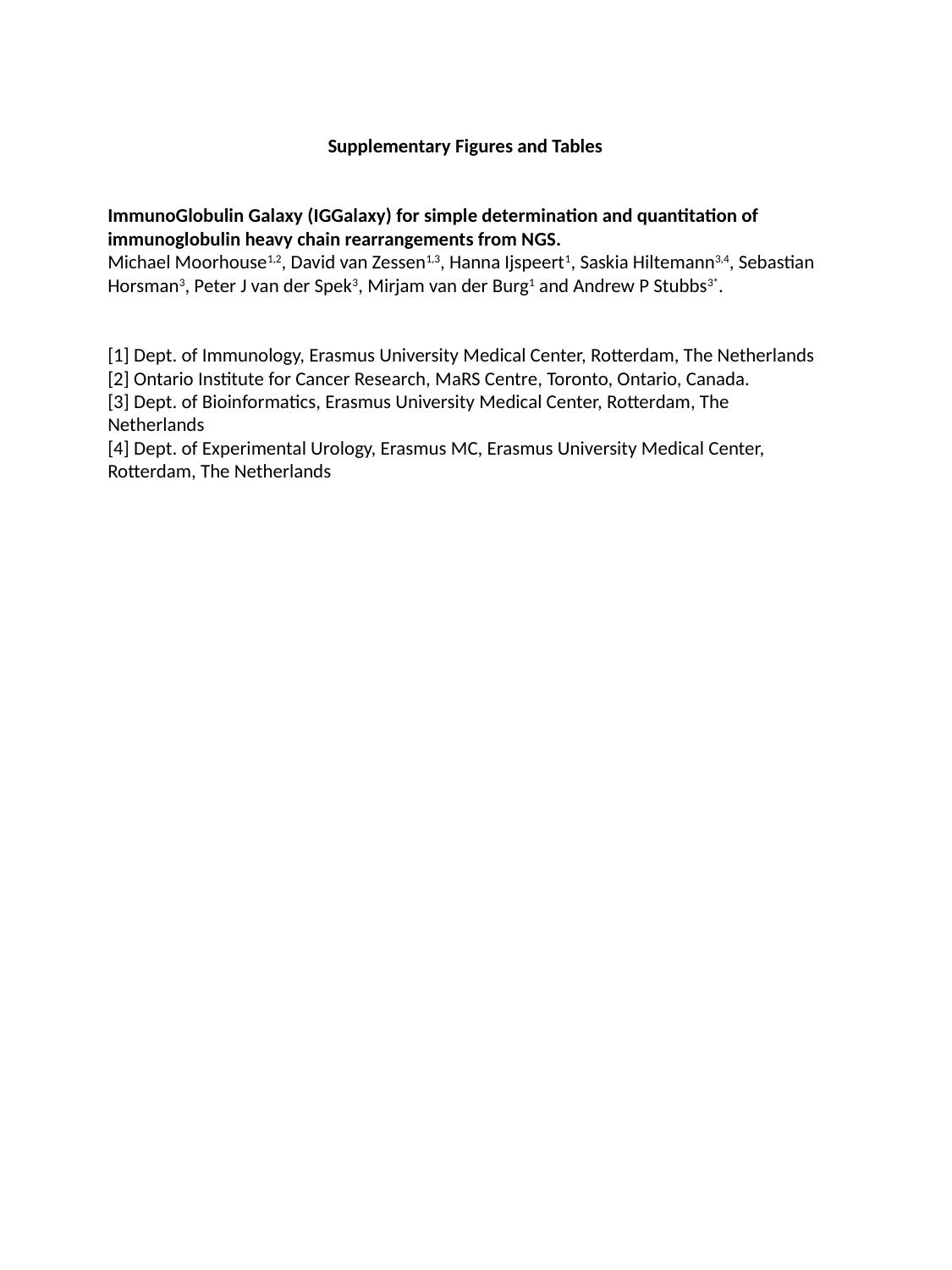

Supplementary Figures and Tables
ImmunoGlobulin Galaxy (IGGalaxy) for simple determination and quantitation of immunoglobulin heavy chain rearrangements from NGS.
Michael Moorhouse1,2, David van Zessen1,3, Hanna Ijspeert1, Saskia Hiltemann3,4, Sebastian Horsman3, Peter J van der Spek3, Mirjam van der Burg1 and Andrew P Stubbs3*.
[1] Dept. of Immunology, Erasmus University Medical Center, Rotterdam, The Netherlands
[2] Ontario Institute for Cancer Research, MaRS Centre, Toronto, Ontario, Canada.
[3] Dept. of Bioinformatics, Erasmus University Medical Center, Rotterdam, The Netherlands
[4] Dept. of Experimental Urology, Erasmus MC, Erasmus University Medical Center, Rotterdam, The Netherlands

## Slide 2
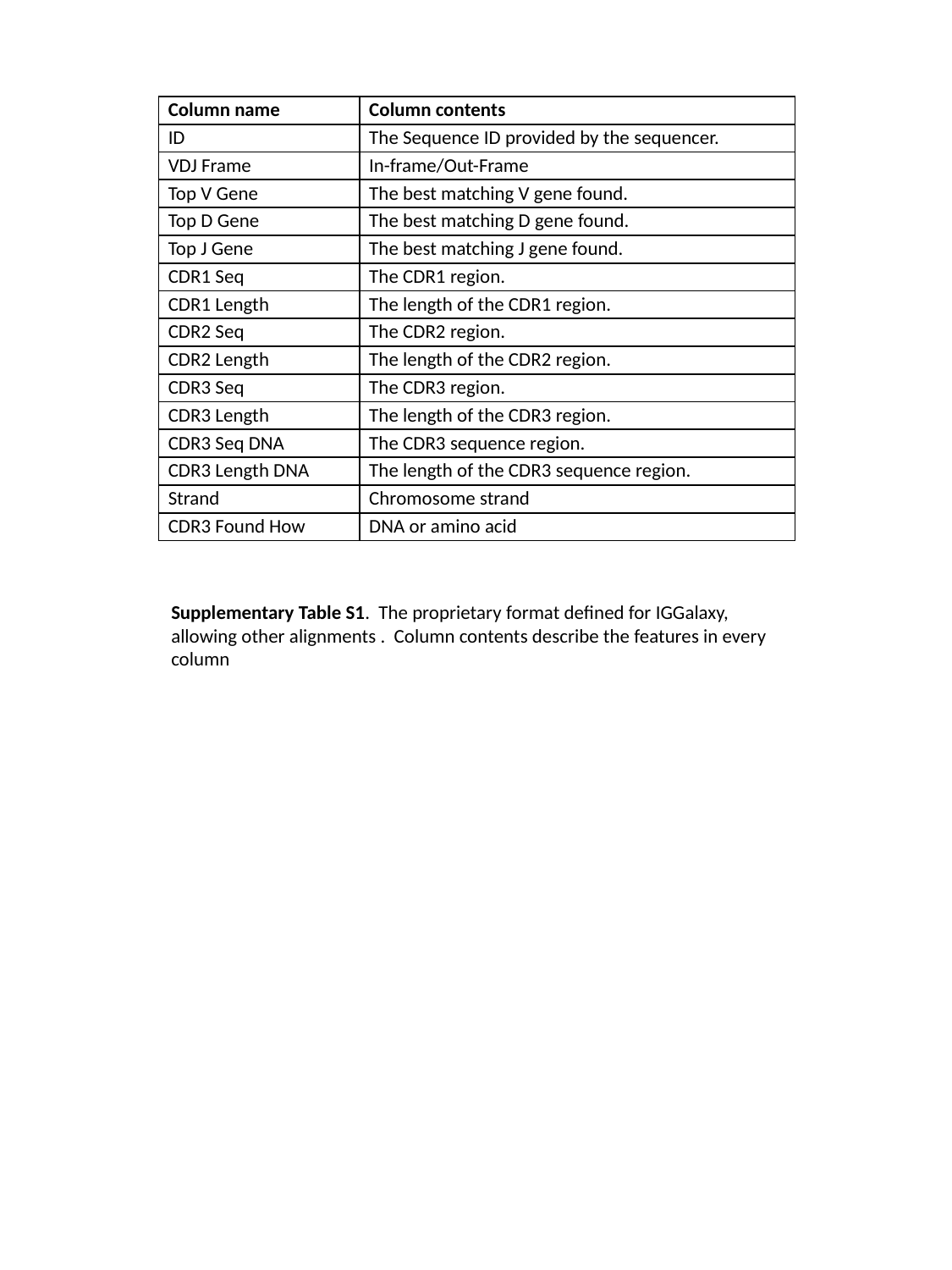

| Column name | Column contents |
| --- | --- |
| ID | The Sequence ID provided by the sequencer. |
| VDJ Frame | In-frame/Out-Frame |
| Top V Gene | The best matching V gene found. |
| Top D Gene | The best matching D gene found. |
| Top J Gene | The best matching J gene found. |
| CDR1 Seq | The CDR1 region. |
| CDR1 Length | The length of the CDR1 region. |
| CDR2 Seq | The CDR2 region. |
| CDR2 Length | The length of the CDR2 region. |
| CDR3 Seq | The CDR3 region. |
| CDR3 Length | The length of the CDR3 region. |
| CDR3 Seq DNA | The CDR3 sequence region. |
| CDR3 Length DNA | The length of the CDR3 sequence region. |
| Strand | Chromosome strand |
| CDR3 Found How | DNA or amino acid |
Supplementary Table S1. The proprietary format defined for IGGalaxy, allowing other alignments . Column contents describe the features in every column

## Slide 3
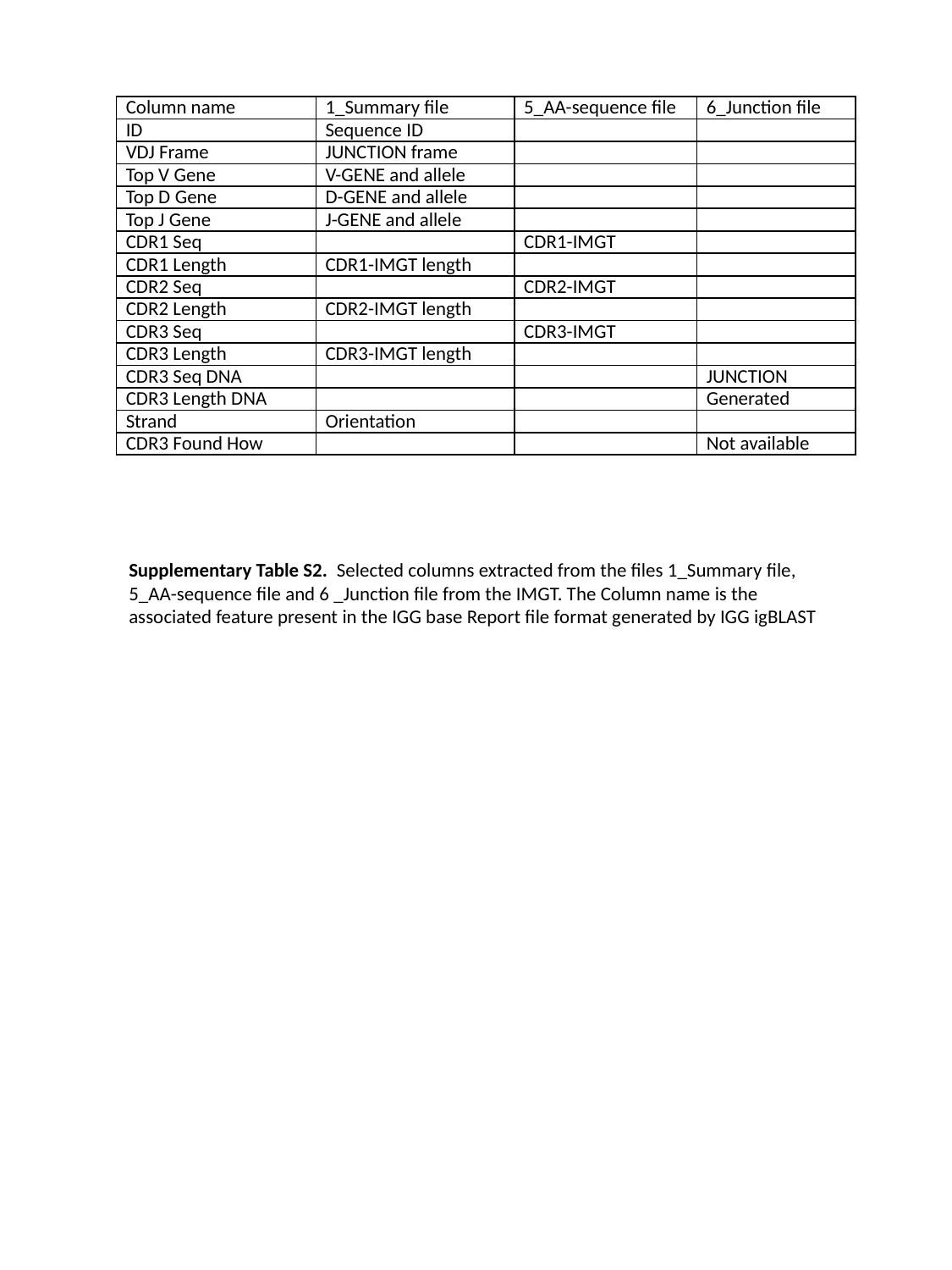

| Column name | 1\_Summary file | 5\_AA-sequence file | 6\_Junction file |
| --- | --- | --- | --- |
| ID | Sequence ID | | |
| VDJ Frame | JUNCTION frame | | |
| Top V Gene | V-GENE and allele | | |
| Top D Gene | D-GENE and allele | | |
| Top J Gene | J-GENE and allele | | |
| CDR1 Seq | | CDR1-IMGT | |
| CDR1 Length | CDR1-IMGT length | | |
| CDR2 Seq | | CDR2-IMGT | |
| CDR2 Length | CDR2-IMGT length | | |
| CDR3 Seq | | CDR3-IMGT | |
| CDR3 Length | CDR3-IMGT length | | |
| CDR3 Seq DNA | | | JUNCTION |
| CDR3 Length DNA | | | Generated |
| Strand | Orientation | | |
| CDR3 Found How | | | Not available |
Supplementary Table S2. Selected columns extracted from the files 1_Summary file, 5_AA-sequence file and 6 _Junction file from the IMGT. The Column name is the associated feature present in the IGG base Report file format generated by IGG igBLAST
